# Mitigating the Harmful Impact of Ageism among Older Individuals: The Buffering Role of Resilience Factors

**DOI:** 10.3390/geriatrics9010001

**Published:** 2023-12-20

**Authors:** Lotte P. Brinkhof, Sanne de Wit, Jaap M. J. Murre, K. Richard Ridderinkhof

**Affiliations:** 1 Department of Psychology, Faculty of Behavioural and Social Sciences, University of Amsterdam, 1018 WS Amsterdam, The Netherlandsk.r.ridderinkhof@uva.nl (K.R.R.); 2 Centre for Urban Mental Health, University of Amsterdam, 1018 WS Amsterdam, The Netherlands; 3 Amsterdam Brain & Cognition (ABC), University of Amsterdam, 1018 WS Amsterdam, The Netherlands

**Keywords:** successful aging, perceived negative ageism, resilience, mental health

## Abstract

Frequent exposure to ageism has significant repercussions on the quality of life and mental well-being/health of older adults. Resilience may play a crucial role in mitigating these effects. The current study aimed to investigate the potential buffering roles of two types of coping variables—behavioral coping and a positive appraisal style—in older adults (N = 2000, aged 55–93). Confirming previous findings, higher levels of perceived negative ageism (PNA) were associated with diminished quality of life and mental well-being, increased depression and loneliness. However, individuals that tend to employ behavioral coping strategies when confronted with challenging/stressful situations showed a weaker relationship between PNA and quality of life, mental well-being, and depression. Embracing a positive appraisal style attenuated the negative impact of PNA on feelings of depression and loneliness. Interestingly, younger older adults appeared to benefit the most from these resilience factors. Despite considerable inter-individual variability, encouraging the utilization of behavioral coping strategies and nurturing a positive appraisal style could serve as effective approaches to mitigate the detrimental effects of PNA.

## 1. Introduction

Throughout the course of our lives, we are constantly exposed to negative societal views and stereotypes about older adults, from subtle ageist jokes in conversations to instances in which individuals are labeled as burdensome or unproductive members of society. These perspectives are assimilated and give rise to explicit and implicit assumptions about aging, older individuals, and what it means to be old [1,2,3]. These assumptions form the foundation of ageism: discrimination, stereotyping, or prejudices against older individuals (even among themselves) based solely on age [4]. Ageism has become a growing global concern as repeated exposure to ageism can have serious negative consequences for older individuals’ self-perceptions of their aging process [5,6] (e.g., through the internalization of stereotypes) and, subsequently, their overall quality of life and mental well-being, as well as depressive symptomatology and feelings of loneliness (e.g., [7,8,9,10,11,12,13]).

However, not everyone seems to be equally affected by ageism [14]. Resilience, the ability to effectively cope with and adapt to difficult or challenging life experiences [15], may play a critical role in mitigating or even neutralizing the detrimental impact of ageism. Yet it is relatively unknown which resources or factors may provide such protection and render some individuals better equipped than others to deal with the challenges of perceiving and experiencing ageism. Gaining such insights is crucial for identifying who is the most vulnerable and for determining how older adults may (be supported to) safeguard themselves against the harmful impact of ageism. To address this knowledge gap, the present study examined to what extent two factors related to coping as fundamental aspects of resilience—behavioral coping and a positive appraisal style—could buffer the relationship between perceived negative ageism (i.e., the stressor) and older individuals’ quality of life and mental well-being, as well as their levels of depression and loneliness. We also explored several resilience-related factors (e.g., self-efficacy, social participation; see the Supplementary Materials, Supplement S1). Unlike behavioral coping and a positive appraisal style, which pertain to immediate specific responses/mechanisms to deal with stressors, these additional factors are thought to operate at a broader and more generalized level, influencing a person’s overall ability to manage stress effectively.

Several systematic reviews and meta-analyses have identified resilience as a factor with significant protective potential for the (mental) health of older adults (e.g., [16]). However, to date, only a limited number of studies have sought to ascertain which specific factors may provide such resilience (or a buffering effect) against the negative effects of ageism (for a review of all studies before 2022, see [17,18,19]). Many of these studies considered resilience factors as mediators. This allows for an examination of (1) the extent to which ageism weakens potential resilience factors (e.g., the subjective ability to bounce back after stressful life-events [19]), thereby compromising psychological well-being; and (2) the extent to which ageism enhances certain aspects that can positively impact mental health (e.g., stronger group identification [9]), thus shedding light on the processes through which certain factors may dampen the negative effects of ageism. However, these approaches do not elucidate the resources that may alleviate or neutralize the harmful effects of ageism. Hence, adopting a moderation design in which resilience factors act as adequate stress moderators that are not necessarily affected by ageism directly seems more opportune for the current aim and will therefore be the focus of this study. 

Some studies have evaluated resilience-related moderators of ageism—(mental) health association, including self-esteem [18]; sense of control [20]; optimism [21]; and positive self-perceptions of aging and/or feeling younger [22]. However, as of yet, the potential moderating effect of adequate coping, which is instrumental to an individual’s ability to adapt and rebound from adversity, in relation to perceived ageism has received minimal attention. Coping encompasses both behavioral and cognitive strategies employed by individuals to manage internal and external demands that are appraised as taxing or exceeding personal resources [23,24]. Behavioral coping strategies include instrumental support seeking, emotional support seeking, the venting of emotions, and planning or acting out (i.e., devising a plan of action/considering which steps to take). These strategies provide individuals with tangible and actionable approaches to dealing with stressors and have been recommended for preventing stress-related issues associated with aging and promoting successful aging [25]. Within the realm of cognitive coping, different strategies can be identified based on their underlying cognitive processes and goals. Some of these strategies are specifically oriented toward positive appraisal and involve cognitive processes that promote positive interpretations and evaluations of stressors (e.g., through positive reappraisal, humor, acceptance, and putting things into perspective or by refocusing on planning [26]). Individuals who tend to appraise potentially threatening or stressful situations in a positive way are thought to have a positive appraisal style. A positive appraisal style can help individuals reframe stressors as manageable challenges and is considered a crucial factor in mitigating the association between stressor exposure and increased levels of psychological distress or mental health problems [27].

Several behavioral and cognitive coping strategies have previously been suggested to forestall the negative effects of perceived racism (or racial discrimination) on (mental) health [28]; similarly, examining the role of coping as potential moderator of the negative effects of perceived ageism seems highly relevant. Therefore, in a large sample of older adults (N = 2000), we investigated whether the tendency to adopt behavioral coping strategies and embrace a positive appraisal style could buffer against the adverse effects of ageism on quality of life, mental well-being, depression, and loneliness (Box 1). This may reveal valuable insights for interventions and personal efforts aimed at shielding individuals from the harmful impact of ageism.

Box 1 **Outcome Variables of Interest.**

Quality of life refers to the cognitive appraisal of one’s life, while mental well-being reflects the emotional response to what life is like [29]. Depression, a common mental health disorder, is characterized by persistent feelings of sadness, hopelessness, and a loss of interest or pleasure in activities [30]. Loneliness, on the other hand, refers to a subjective feeling of social isolation or a lack of meaningful connections with others [31]. Although both quality of life and mental well-being are influenced by depressive symptoms and loneliness, these mental-health-related variables also exhibit distinct characteristics. By evaluating these outcome variables separately, we can inform more tailored interventions to support older individuals in navigating the challenges posed by ageism.

Individuals who tend to adopt behavioral coping strategies when dealing with stressful or challenging experiences may be better equipped to deal with ageism-related distress. Behavioral coping strategies could enable individuals to question the validity of ageist beliefs directed at them (e.g., by discussing the experience with a friend) and reduce negative feelings about oneself. Similarly, those who have a positive appraisal style may be more likely to mitigate or neutralize their negative thoughts derived from negative ageism experiences, thereby reducing the chance that ageism experiences will exert an enduring impact on their quality of life and mental well-being and/or will cause heightened levels of depressive symptomatology or feelings of loneliness. Both the use of behavioral coping strategies and the extent to which one positively appraises stressful situations have been claimed to be modifiable [27,32,33,34] and may therefore provide opportune target points for interventions or personal efforts aimed at promoting adequate coping with negative ageism experiences. 

The benefits of these coping styles may also vary with age. While the level of perceived ageism tends to increase with age [7,35], an increasing number of studies suggest that ageism may be more harmful to relatively younger seniors (e.g., [12,36,37]). This has been attributed to the fact that ageism becomes more self-relevant in middle age and that facing ageism may have been neglected as a remote future experience and even serves as a “rude awakening” that conflicts with individuals’ (positive) self-image and expectations of the future, thereby causing the most distress during this life phase [36,38]. In contrast, older-older adults may be more likely to accept or have already internalized age-related stereotypes [39] and/or even perceive ageism as legitimate [40]. Moreover, older age could offer some protection against the harmful effects of ageism as older adults may have developed a larger repertoire of coping strategies from dealing with previous experiences of ageism, which provides resilience for subsequent exposure [41]. Altogether, it seems plausible that especially those younger in age may benefit from high levels of the protective resilience factors of interest; therefore, the role of age in moderating the potential benefits of the resilience factors of interest was also examined. 

Specifically, in this study, we set out to test several hypotheses. First, we hypothesized that there would be a negative/unfavorable relationship between perceived negative ageism and quality of life and mental well-being, depression, and loneliness. Second, we examined the moderating role of age in these associations, anticipating weaker links for older individuals. Third, we expected a neutralizing/buffering effect of behavioral coping and a positive appraisal style on the relationship between perceived negative ageism and the outcome variables of interest. Finally, we hypothesized that age moderates the buffering/neutralizing effect of these resilience factors such that especially those younger in age were expected to benefit from high levels of resilience factors.

## 2. Methods

### 2.1. Sample Characteristics

Participants were drawn from a larger pool of older individuals participating in an ongoing online study on successful aging and resilience in the Netherlands (www.seniorendoenmee.nl). This online study used a battery of questionnaires and tests that assessed various relevant factors across different domains, such as physical, psychological, cognitive, social, and environmental factors (approved by the local ethics committee of the University of Amsterdam, 2020-DP-12556). To be eligible for enrolment in the study, participants had to be 55 years or older, living in the Netherlands, and not have a diagnosis of dementia. This relatively broad age range was adopted to accommodate the considerable individual differences in the aging process, as well as to adequately examine the role of age in moderating the potential benefits of the resilience factors of interest. Other exclusion criteria included inadequate proficiency with the Dutch language, impaired vision, or an inability to independently use a computer or laptop to perform tasks such as clicking a mouse and typing on a keyboard. The first 2000 participants (1334 females, 665 males, and 1 other (one participant chose the option “other” when indicating their gender identity, expressing a preference not to identify as exclusively male or female); age: *M =* 68.3, *SD =* 6.99, 55–93) that completed the full battery of questionnaires and tests within 14 days were included in this analysis. The majority of participants were highly educated (86%, category 6 or 7 of the Dutch Verhage scale [42]) and resided in strongly to extremely urbanized areas (n = 1318; not urbanized to moderately urbanized, n = 671; no data, n = 11). This is a postal-code-inferred measure of population density, quantified as the average number of addresses per km² within a circle with a radius of 500 m from the geographical center of an individual’s postal code area (as obtained from the Geoscience and health cohort consortium project [43]). The level of urbanization was categorized as follows: extremely urbanized, 2500 surrounding addresses or more; strongly urbanized, 1500 to 2500 surrounding addresses; moderately urbanized, 1000 to 1500 surrounding addresses; hardly urbanized, 500 to 1000 surrounding addresses; and not urbanized, fewer than 500 surrounding addresses. For 11 participants, no reliable postal code was provided. The level of perceived negative ageism was similar across all urbanization groups, F (1, 1987) = 1.31, *p* > 0.05). 

### 2.2. Materials

#### 2.2.1. Perceived Ageism

The 5-item Perceived Negative Ageism subscale of the 8-item Perceived Ageism Questionnaire (PAQ-8) was used to assess the level of PNA [7]. The PNA subscale comprises five items reflecting negative forms of ageism (e.g., being addressed as a child because of one’s age; α: 0.81). Each of the items describes different situations or attitudes that older adults may have experienced or encountered in the past year. Participants were asked to report how often each situation had occurred using a 5-point Likert Scale (1 = never, 2 = barely, 3 = sometimes, 4 = often, and 5 = very often). Item scores were summed to a total ranging from 5 to 25, with higher scores indicating higher levels of PNA. 

#### 2.2.2. Quality of Life

The World Health Organization’s Quality of Life (WHOQOL)-OLD [44] (α: 0.87) assessment was used to assess quality of life based on six subscales, each containing four items: (1) sensory abilities (e.g., “To what extent do impairments to your senses affect your daily life?”), (2) autonomy (e.g., “How much freedom do you have to make your own decisions?”), (3) satisfaction with past, present, and future activities and achievements in life (e.g., “How satisfied are you with what you have achieved in life?”), (4) social participation (e.g., ““To what extent do you feel that you have enough to do each day?”), (5) concerns, worries and fears about death and dying (e.g., “How scared are you of dying?”), and (6) the ability to have personal and intimate relationships (e.g., “To what extent do you feel a sense of companionship in your life?”). All 24 items were rated using a 5-point Likert Scale with different wordings and summed to a total quality of life score. To ensure that higher quality of life scores reflected a better quality of life, some items were reverse-scored prior to summation (24–100).

#### 2.2.3. Mental Well-Being

The 14-item Warwick Edinburgh Mental Wellbeing Scale (WEMWBS [45]; α: 0.89) was used to measure MWB. All 14 items address positive aspects of mental health (e.g., “I’ve been feeling relaxed”) and were scored on a 5-point Likert scale (1 = never, 2 = barely, 3 = sometimes, 4 = often, and 5 = always). Items were summed to a total ranging from 14 to 70, with higher scores indicating better MWB.

#### 2.2.4. Depression 

The Centre of Epidemiological Studies Short Depression Scale (CES-D-10 [46]; α = 0.82) was used as a measure of depressive symptomatology in the preceding week. Each item (e.g., “I was bothered by things that usually don’t bother me”) was rated on a 4-point scale ranging from 0 (less than one day) to 3 (5–7 days), and these scores were summed to a total depression score (0–30). Prior to summation, two positively formulated items were reverse-scored. Higher scores indicated more depressive symptoms.

#### 2.2.5. Loneliness

The 11-item Loneliness Scale (LS), as developed by de Jong-Gierveld and van Tilburg [47], was used to assess overall levels of loneliness (α = 0.87). The scale consists of a total 11 statements, with six phrased negatively (emotional subscale, e.g., “I miss having a really close friend”) and five phrased positively (social subscale, e.g., “There is always someone I can talk to about my day-to-day problems”). Participants were asked to rate each item using the following response options: “yes!”, “yes”, “more or less”, “no”, and “no!”. A total loneliness score was calculated by adding the number of neutral and positive answers on the positively formulated items to the number of neutral and negative answers on the negatively worded items. Higher-sum scores (0–11) reflected greater loneliness.

#### 2.2.6. Behavioral Coping and Positive Appraisal

The Positive Appraisal Style Scale and the Behavioral Coping Scale were used to measure positive appraisal style and behavioral coping, respectively [48]. The 14-item PASS reflects positive appraisal content and processes and includes one subscale of the brief COPE [49] (i.e., humor; two items), five subscales of the CERQ short [50] (i.e., positive reappraisal, acceptance, putting things into perspective, refocusing on planning, and positive refocusing; two items per subscale), and two self-generated items (related to distanced stressor appraisal). The BCS constitutes 8 items from the brief COPE, covering four different subscales: the use of instrumental support, emotional support seeking, the venting of emotions, and planning/acting out (two items each). Participants completed the relevant items of the brief COPE and CERQ short, as well as the two self-generated items. All selected items of the brief COPE (including “I’ve been getting emotional support from others”) were scored on a 4-point Likert Scale (1 = not at all, 2 = a little bit, 3 = quite a lot, and 4 = a lot), and the other 12 items (e.g., “I think that I have to accept that this has happened”) were scored on a 5-point Likert scale (1 = (almost) never, 2 = sometimes, 3 = regularly, 4 = often, and 5 = (almost) always). The items from the subscales of the BCS were summed to a total behavioral coping score ranging from 8 to 32 (α = 0.79). A positive appraisal style score was determined by taking the average of the z-normalized scores of the items derived from the brief COPE, the short CERQ, and the self-generated items (α = 0.86).

### 2.3. Data Analysis 

**Figure 1:**
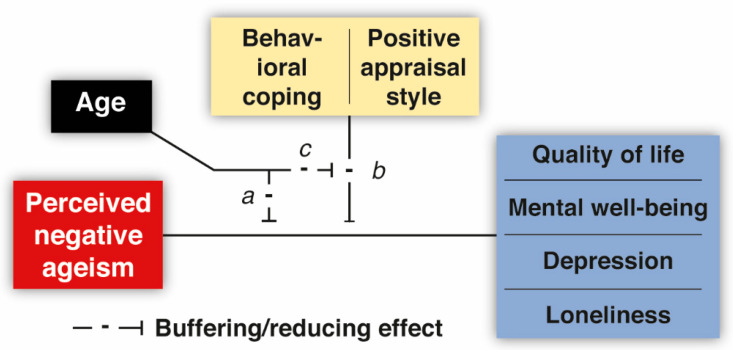
Overview of the tested moderation models of perceived negative ageism. Note: For each outcome variable of interest (blue), a total of two moderation models were adopted to evaluate the buffering role of each of the two resilience factors of interest (yellow) separately. Path *a*: the moderating role of age in the relationship between PNA and each outcome variable. Path *b*: the buffering role of the resilience factors in the relationship between PNA and the outcome variables. Path *c*: the moderating role of age in the buffering effect of the resilience factors.

All analyses were conducted in R 4.1.2. [51], with alpha set at 0.05 per default. For all variables, the sampling distribution was assumed to be normal due to the large sample size. Initially, we computed bivariate correlations among the study variables of interest for descriptive purposes and to establish whether perceived negative ageism was indeed related to more negative outcomes on quality of life, mental well-being, depression, and loneliness. Subsequently, for each outcome variable, a simple two-way regression model was adopted in which the moderating role of age (i.e., the interaction term) of the relationship between perceived negative ageism and each outcome variable was established (see Figure 1, path *a*). Next, a series of more complex multiple regression models was employed to establish to what extent the two resilience factors of interest (i.e., behavioral coping and positive appraisal) can buffer the detrimental association between perceived negative ageism and the outcome variables (see Figure 1, path *b*). To this end, each outcome variable was included in two different models (8 moderation analyses in total), with each of these models including the two-way interaction between the level of perceived negative ageism and one of the resilience factors of interest as a predictor. Moreover, age was also added as moderator of this two-way interaction (see Figure 1, path *c*). If this three-way interaction term was insignificant, we assumed that the moderating effect of the resilience factor was not affected by age, and the analysis was conducted again with age included as an independent predictor only [52]. (failure to remove a higher-order interaction term if this is not significant can lead to incorrect conclusions that there is no lower-order moderation effect). To interpret significant moderation terms, the emtrends function of the emmeans package [53] was used to conduct a simple slope analysis at two levels of the resilience factors (extremes: low and high) and five levels of age (extremes and quartiles: 55, 63, 68, 73, and 93). This allowed us to infer how the associations between perceived negative ageism and the outcome variables changed as a function of age in small steps. Estimated marginal trends were Bonferroni-corrected. 

To gain meaningful insights into the significance of our findings, we extended beyond merely reporting effect size and incorporated a robustness bootstrapping analysis (see, e.g., [54]). In this rigorous procedure, we randomly selected 500 participants from the full sample and temporarily excluded them, creating a new subsample of 1500 participants. Subsequently, we subjected the remaining dataset to a moderation analysis, as previously described. This bootstrapping process was repeated 1000 times, generating a multitude of subsamples, and a moderation analysis was conducted for each. In doing so, we were able assess how frequently a two-way interaction, a three-way interaction, or no moderation effect was observed (using an alpha level of 0.05) and thereby obtain a comprehensive understanding of the stability of the results. 

A similar procedure was used to explore the potential of a number of other resilience-related factors, including general self-efficacy [55,56,57,58,59,60,61], self-esteem [18,62,63], social participation [64,65,66,67,68,69,70,71], and the big five personality traits [72,73,74,75,76,77]. Results pertaining to these factors will solely be reported in the Supplementary Materials, Supplement S1, but relevant findings will be highlighted in the discussion.

We also conducted a post hoc analysis to explore the influence of gender, differentiating between male and female participants. Here, we again used the bootstrapping procedure as described previously. However, to ensure a sufficiently large sample within each bootstrap, 85% of the respective subgroup’s total size was included (males: 665 × 0.85 = 565; females: 1334 × 0.85 = 1134). Results pertaining to this post hoc analysis are reported in the main text but will only be evaluated in terms of potentially intriguing nuances that may warrant further investigation, given the exploratory nature of this analysis. 

## 3. Results

A summary of the statistics as well as the bivariate correlations among the variables of interest are shown in Table 1. These results confirm the unfavorable associations between the level of PNA and individuals’ quality of life and mental well-being/health. 

### 3.1. Moderation

**Figure 2:**
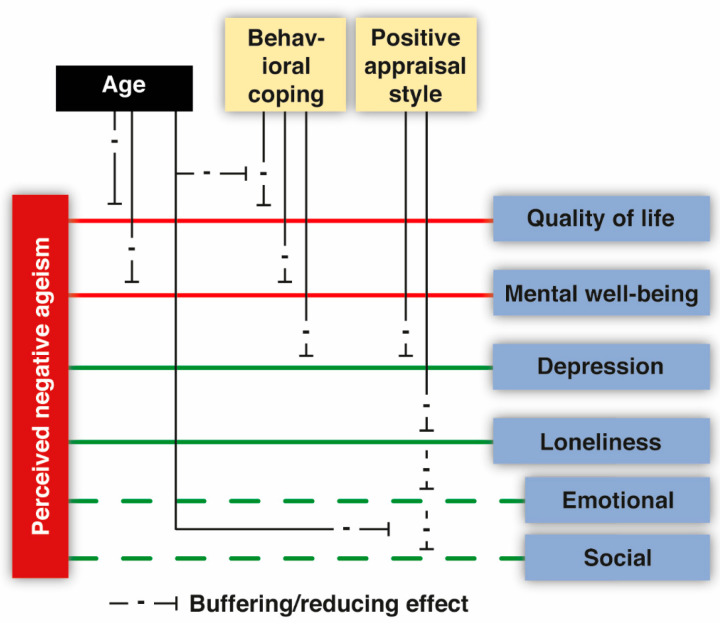
Results of moderation models of perceived negative ageism. Note: the effects reported for emotional and social loneliness correspond to an exploratory analysis and are shown with dashed lines.

To better understand the potential moderating effects of age on the buffering effects of the proposed resilience factors, an initial analysis was conducted to determine if the association between PNA and quality of life and mental well-being, as well as depression and loneliness, was moderated by age. As expected, the negative relationship between PNA and both quality of life and mental well-being became less pronounced as age increased (see Figure 2; statistics are summarized in Table S3 of Supplement S2 in the Supplementary Materials). While a comparable age-related pattern was evident for both depression and loneliness, the findings did not reach statistical significance. This suggests that the harmful association between PNA and these variables was consistent across all age groups.

**Figure 3:**
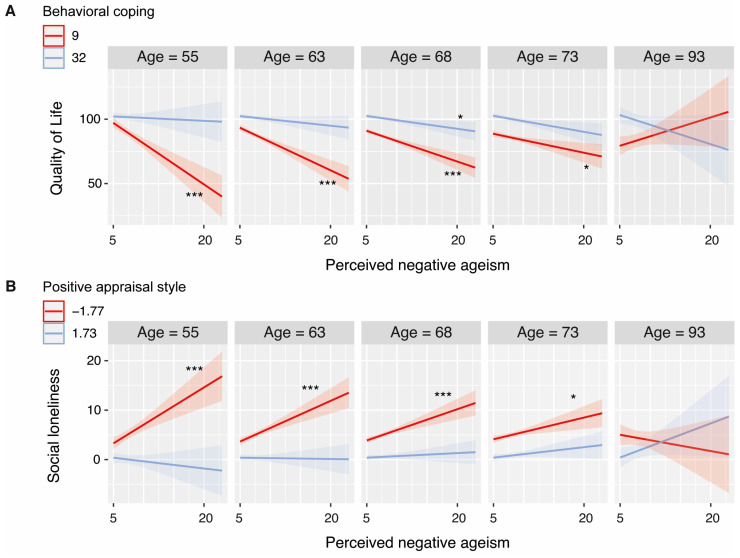
Simple slopes of three-way interaction models. Note: Illustrated patterns should be interpreted with caution as we observed a relatively large proportion of failed moderations and/or two-way interactions in our bootstrapping analysis. * *p* < 0.05 and *** *p* < 0.001. The *p*-values of the simple slopes are Bonferroni-corrected.

Behavioral coping appeared to be the strongest buffering factor in mitigating the negative association between PNA and quality of life, mental well-being, and depression but not loneliness (see Figure 2; statistics are summarized in Table 2). These findings were relatively robust, as indicated by minimal counts of failed moderation in the bootstrapping analysis (see Table 3). For mental well-being and depression, a relationship with PNA was only observed among those with a low tendency to use behavioral coping strategies when dealing with stressful or challenging situations (i.e., full moderation). For quality of life, a similar benefit of behavioral coping was observed, but this also seemed to depend on age. That is, the strength of the association with PNA was moderated by behavioral coping, except for those relatively high in age. Thus, the buffering effect of behavioral coping seemed to decrease with age. This effect was mainly driven by the fact that the youngest individuals showed a particularly strong relationship between PNA and quality of life at low levels of behavioral coping (see Figure 3). While this moderation effect of age was found for the majority of bootstraps, we also observed a relatively large proportion of failed moderations (see Table 3); thus, this finding should be interpreted with caution. 

Embracing a positive appraisal style appeared to be a promising age-independent buffer for the relationship between PNA and both depression and loneliness but not for the other outcome variables of interest (see Figure 2; statistics are summarized in Table 4). For depression, this finding was not highly robust (see Table 3): while moderations were found for the majority of bootstraps, a relatively large proportion of failed moderations was observed. An exploratory post hoc analysis revealed that having a positive appraisal style is helpful for mitigating the negative effects of PNA on emotional loneliness for all ages (see Figure 3). For social loneliness, positive appraisal style may only be beneficial among the youngest age groups, although a substantial proportion of two-way interactions or failed moderations was found as well (see Table 3).

### 3.2. Post Hoc Analysis: The Influence of Gender

Our post hoc analysis concerning the role of gender revealed some interesting nuances (see Table 5, Table 6 and Table 7). It seems that age moderates the relationship between PNA and quality of life as well as mental well-being exclusively among females. Hence, PNA seems to exhibit consistent negative effects on quality of life and mental well-being among men, regardless of age. Furthermore, the potential buffering roles of behavioral coping and positive appraisal styles appear more pronounced among females while offering relatively fewer advantages for males. An exception emerges in the case of quality of life, for which the benefits for females appear somewhat constrained. Notably, certain effects observed within the full sample did not persist within any of the gender-specific subgroups, suggesting that in some instances, these effects may be influenced by statistical power. Indeed, for these cases, a considerable proportion of two-way interactions was identified among females.

## 4. Discussion

Frequent exposure to ageism can have negative implications for older individuals’ (aged 55 years or older) quality of life, mental well-being, and mental health. However, individual responses to perceived ageism vary widely, suggesting that individual differences in resilience may play a crucial role. In line with the notion that adequate coping equips individuals with the skills, mindset, and emotional strength to effectively face and navigate adversity, the present study suggests that adequate coping can alleviate and sometimes even neutralize the negative effects of perceived negative ageism (PNA), especially among older adults who are relatively young. Specifically, we found that behavioral coping mitigated the negative effects of PNA on quality of life, mental well-being, and depression but not loneliness. A positive appraisal style buffered the relationship between PNA and both depression and loneliness. The most important findings and their relevant nuances are discussed below. 

### 4.1. Ageism Seems Particularly Harmful for Those Younger in Age

Although the level of PNA increased with age [7,35], the negative relationships between PNA and both quality of life and mental well-being became less pronounced with increasing age. This is in line with our hypothesis and numerous previous studies (e.g., [12,36,37]), and concurs well with the idea that ageism may be particularly harmful when it becomes self-relevant for the first time [36,38]. Moreover, it aligns with earlier suggestions that older-older adults may have assimilated and internalized age-related stereotypes [39] and may even perceive ageism as legitimate [40]. In addition, or alternatively, these findings could be driven by older individuals being better equipped to navigate ageism because they have developed knowledge and adaptive coping strategies throughout their life [78] other than those specified as behavioral coping or a positive appraisal style in the present study (e.g., finding or creating meaning and purpose in challenging or difficult situations, being assertive in expressing needs and boundaries, and/or critical thinking to challenge stereotypes and advocate for fair treatment). Surprisingly, no moderating effects of age surfaced in relation to depression and loneliness, implying that high levels of perceived ageism contribute to higher levels of depression and loneliness independent of age. Given that the level of PNA increased with age, it remains evident that older-older adults may be less severely affected by PNA, but the negative consequences for their mental health are likely not entirely averted. Age also moderated the extent to which behavioral coping and a positive appraisal style buffered the negative relationship between PNA and some outcome variables. These findings will be discussed in the next section. 

### 4.2. Behavioral Coping

Behavioral coping appears to be a promising factor in mitigating the negative effects of ageism. Indeed, previous research on discrimination and health already suggested that coping behaviors can moderate the link between perceived racial discrimination and (mental) health [28]. The present study adds to our understanding and shows that behavioral coping behaviors may also protect individuals from ageism effects specifically. The potential moderating effect of one specific adaptive behavioral coping strategy, namely seeking social support/advice from family, relatives, and friends, was previously examined by Kim and colleagues [79]. They reported beneficial effects of this behavioral coping strategy in mitigating the negative relationship between emotional responses caused by ageism experiences and depression. Our findings extend these results and show that other behavioral coping strategies may also prove to be effective, not only for neutralizing PNA’s effects on depression but also for quality of life and mental well-being. 

Interestingly, for quality of life, the potential of behavioral coping as resilience factor seemed to decrease with age, which suggests that younger individuals in particular could benefit from seeking instrumental or emotional support, venting their emotions, and planning or acting out. This can be explained by the fact that while PNA is encountered more frequently at higher ages, the detrimental consequences of PNA decrease with age, rendering additional efforts in the form of coping less imperative (note that both behavioral coping and a positive appraisal style were negatively associated with age). Additionally, it may, at least partially, be explained by the fact that the deployment of behavioral coping strategies often requires the involvement of others, while social networks tend to decline with age [80]. In support of this, we found that behavioral coping was more strongly associated with age than positive appraisal style was. Hence, younger individuals may benefit from larger social networks they can resort to, enabling them to use behavioral coping as a strategy to deal with ageism distress and thereby protecting their quality of life. Why this is particularly true for quality of life remains an important question. While the effect of PNA on mental well-being also seemed to decrease with age, behavioral coping appeared to neutralize PNA’s negative relationship with mental well-being similarly across all age groups. 

While behavioral coping seemed to buffer PNA’s effects on depression, feelings of loneliness were not harnessed by these strategies. In line with previous argumentations and the observation that greater feelings of loneliness were associated with low levels of behavioral coping, behavioral coping may be less effective at protecting against increases in loneliness if a reduction in social connections prevents one from employing behavioral coping as useful strategy. Moreover, we may speculate that behavioral coping strategies are less effective in buffering ageism’s impact on loneliness if individuals resort to their social network but subsequently encounter social interactions that are characterized by rejection, exclusion, or a lack of understanding. This speculation calls for deeper investigation, including regarding the reasons why this pattern does not seem to hold true for quality of life and depression.

Altogether, these findings lend support to our hypotheses concerning the buffering/neutralizing impact of behavioral coping on perceived negative ageism’s influence on quality of life, mental well-being, and depression, albeit not on loneliness. While younger individuals seem to benefit more strongly from high levels of behavioral coping, the evidence concerning this hypothesis is less conclusive across all outcome variables.

### 4.3. Positive Appraisal Style

A positive appraisal style mitigated the harmful effects of ageism on depression and loneliness. This is in line with an earlier study reporting the benefit of optimism in moderating the ageism–psychological distress link [21] and suggests that this resilience factor is particularly beneficial in protecting against two of the most common mental health issues in later life. Indeed, positive appraisal concerns the (emotional) re-evaluation of a certain situation. Individuals who embrace a positive appraisal style are thought to appraise an adverse event or threat in a realistic or positive way, thereby avoiding catastrophizing, pessimism, and helplessness. When negative ageism is frequently encountered, this may help to prevent or neutralize intrusive and negative thoughts about the consequences of aging, contributing to more favorable mental health outcomes. That is, earlier research reported that those with more negative expectations about the aging process, or even those that associate old age with loneliness in later life, have a higher change of becoming lonely and depressed in the future [81,82]. Appraising ageist encounters in a non-negative way may prevent such negative trajectories (e.g., by reappraising it or putting it in perspective, for instance, by considering it a form of humor or by distancing oneself from ageist encounters by thinking “he/she is wrong to think this applies to me”). 

Interestingly, while a positive appraisal style seemed to buffer the negative effects of PNA on emotional loneliness at all ages, we found preliminary evidence suggesting that the negative impact on feelings of social loneliness may only be neutralized by positive appraisal among relatively young individuals. Emotional loneliness pertains to the subjective experience of lacking intimate and meaningful emotional connections with others. Social loneliness, on the other hand, refers to a perceived absence or insufficiency of social interactions and companionship. These patterns may be explained by the fact that emotional connections remain important throughout the lifespan, while social loneliness may be particularly impactful for relatively young individuals who may place greater value on broad social networks [83]. On the other hand, again, it may be that it is more challenging to alleviate PNA’s effects on social loneliness through positive appraisal as strategies like putting things in perspective or accepting the fact that certain individuals exhibit ageist attitudes may not necessarily lead to a substantial increase in the availability of social interactions—although it may help to reduce negative feelings about the ageist experience. 

Altogether, these findings affirm our hypotheses regarding the buffering/neutralizing impact of a positive appraisal style on perceived negative ageism’s impact on depression and mental well-being, though not on the broader constructs of quality of life and mental well-being. Some instances suggest that age may play a moderating role, but this observation is, again, less conclusive.

### 4.4. Intervention Potential

Encouraging individuals to proactively address ageism-related stressors by seeking instrumental and emotional support, venting their emotions, or planning/acting out, as well as to embrace a positive appraisal style, may help individuals to reduce the toll of ageism and bolster their quality of life, mental well-being, and mental health. Several programs have proven effective in diminishing ageist perceptions among younger and older adults [84]. However, as far as we know, there have been limited attempts to devise and test an intervention program that can help older individuals to cope with ageist experiences and challenge the validity of ageist perceptions directed at them. Jeste, Trechler, and their colleagues [85,86] describe one of the few resilience-promoting interventions for older adults that incorporated explicit discussions on the impact of age discriminations and stereotypes, along with methods to fight those stereotypes and improve perceptions of aging. Current findings suggest it may be promising to develop an intervention program or course that specifically focuses on promoting the use of behavioral coping strategies and cultivating a positive appraisal style as a means of empowering older individuals to mitigate the negative consequences of ageism. Surprisingly, despite previous recognition of coping strategies as possible targets for intervention in various stress-related contexts, there have been relatively few attempts to actively manipulate these factors to instigate real changes. Importantly, those few attempts have yielded promising results: specific behavioral coping strategies were successfully enhanced and positive appraisal style dimensions were fostered within the context of resilience and stress management [34,87,88]. To illustrate this point, positive reappraisal among older individuals with a chronic physical illness was increased after learning how to effectively use this coping strategy, contributing to more positive emotions [87,88]. Hence, it seems opportune for future research to develop and test coping-based ageism interventions and identify by whom such programs may be delivered, and which settings are most appropriate (e.g., health care and community centers or assisted living facilities for seniors). Indeed, health-care professionals seem well-suited to administering such programs, either on an individual basis or as part of a group-based initiative. 

### 4.5. Limitations and Future Directions

Despite these promising findings and the potential benefits of coping programs, it is imperative to acknowledge that not all observations are equally robust. For instance, while we established that age may be an important moderator of the buffering effect of behavioral coping for quality of life, it is worth noting that a relatively high number of bootstrap iterations showed no such moderation. Similarly, for a substantial number of bootstrapping iterations, no moderation effect for positive appraisal style was found for the PNA—depression link. This indicates that these outcomes were notably sensitive to the composition of participants in each subsample, which potentially limits the generalizability of these findings to some extent. This suggests that the patterns of interest are complex and might be contingent upon specific participant characteristics; potentially, the variation stems from factors that have not been accounted for and could imply that some (subgroups) of individuals do benefit from effective coping, whereas others do not. Indeed, our post hoc analysis provided some interesting nuances regarding the influence of gender. Specifically, our findings suggest that females may derive greater benefit from behavioral coping efforts and a positive appraisal style compared with males. Importantly, this discrepancy is not attributable to differences in the level of PNA or to a weaker association between PNA and the outcome variables (although for depression, the link with PNA was notably stronger among females, yet still evident among males). This suggests that on average, males may be more susceptible in the face of ageism and potentially derive fewer advantages from programs aimed at promoting behavioral coping and a positive appraisal style. These findings may be explained by gender differences in the effectiveness of employing these resilience factors. That is, women often experience multiple layers of discrimination due to the intersection of ageism with gender bias [89]. These intersecting identities can lead women to develop enhanced resilience and coping strategies, making them more adept at using behavioural coping and positive appraisal styles when dealing with ageism. Future research may dive further into these subgroup patterns and potential explanations thereof and use a confirmatory study design to replicate current exploratory findings. Furthermore, the role of other factors that could influence the benefits of behavioral coping and a positive appraisal style, including cultural differences, socio-economic status, health conditions, or the presence of other major life stressors, should also be explored. Indeed, the current sample was highly educated, and different patterns may arise when less educated participants are included. 

In any case, the uncertainty of some of our findings emphasizes that the negative effects of ageism may be somewhat resistant, at least to some individuals, and that it can be challenging to mitigate these effects. This conclusion is also supported by the series of exploratory analyses that we conducted. We explored the role of several distal resilience factors, including self-efficacy, self-esteem, social participation, and several personality traits. As reported in the Supplementary Materials, Supplement S1, we did not find convincing evidence for strong buffering effects of any of these factors, suggesting that overall, the effects of negative ageism remain largely exempt from moderation by these distal resilience factors. Altogether, this underscores that despite some promising effects for adequate coping, resilience is not a panacea, and it remains of the utmost importance to combat the sources of ageism in society, as mitigating its effects may not be manageable for everyone. 

The present study reports cross-sectional relationships and identifies the potential protective influence of various resilience factors on a broad time scale. However, perceived ageism may vary on a daily or weekly basis [90]. An ageist encounter on a certain day may activate more negative self-perceptions of aging or lower levels of self-efficacy (or another mediating factor), which could directly impact one’s mental well-being or mood/mental health (e.g., more depressive symptoms). It is of great interest to determine if resilience factors, such as the tendency to use behavioral coping strategies and/or embrace a positive appraisal style, could mitigate these kinds of processes and at what stage(s) they may be able to intervene and/or regulate temporary declines in mood/well-being that could otherwise elevate the risk of escalating toward mental health issues in the long term.

Finally, given the subjective nature of perceived ageism, it is essential to acknowledge that the level of perceived ageism may not always align with the level of ageism that individuals are objectively exposed to. Although older individuals may encounter ageist attitudes, beliefs, and behaviors, they may not necessarily perceive or be aware of them, or they may erroneously perceive a comment as ageist. Such a discrepancy could result in varying levels of perceived ageism among older individuals, even if they are exposed to comparable levels of ageism objectively. Individual differences in (mental) health status or resilience mechanisms may play a crucial role in this context as well. For instance, research suggests that individuals who are depressed may be more likely to perceive high levels of ageism due to their negative outlook on life [91]. Conversely, some resilience factors, such as those evaluated in the current study, may help to protect individuals from *perceiving* ageism, even when they are exposed to it. For instance, individuals who have a positive appraisal style may inherently view the world more positively and thereby not perceive ageism objectively. Exploring these risk and protective factors may provide a comprehensive understanding of the subjective nature of perceived ageism and how to mitigate its impact, as well as reduce it on a societal level. 

### 4.6. Summary

In summary, the current study shows that adequate coping may play a significant role in mitigating the deleterious effects of perceived ageism. Behavioral coping and a positive appraisal style both seem to be opportune target points for interventions to promote resilience against the impact of ageism, at least among older adults with higher education levels and especially among females. Moreover, those younger in age seem to benefit the most. Nonetheless, our findings also highlight that this may not work for every individual and that neutralizing the effects of perceived ageism can be extremely challenging. Even the most resilient older adults may still experience the negative impacts of ageism. Hence, in addition to enhancing resilience among older individuals, it remains highly important to address and ultimately reduce ageism at both individual and societal levels and to create a more equitable and inclusive society for people of all ages.

## Figures and Tables

**Table 1:** Summary of statistics and bivariate correlations.

	M	SD	Min	Max	PNA	MWB	QoL	DEP	LONE	BC	PAS
**PNA**	7.40	2.64	5	23							
**MWB**	55.7	5.89	34	70	−0.19						
**QoL**	94.8	9.59	51	120	−0.32	0.62					
**DEP**	15.9	4.52	10	38	0.22	−0.63	−0.61				
**LONE**	2.71	3.05	0	11	0.24	−0.44	−0.59	0.45			
**BC**	21.6	3.69	9	32	−0.09	0.21	0.26	−0.10	−0.34		
**PAS**	0.05	0.60	−1.77	1.73	−0.06	0.39	0.36	−0.26	−0.24	0.30	
**Age**	68.3	6.99	55	93	0.20	0.02	−0.16	0.00	0.07	−0.15	−0.09

Note: PNA: perceived negative ageism; MWB: mental well-being; QoL: quality of life; DEP: depression; LONE: loneliness; BC: behavioral coping; PAS: positive appraisal style. The bivariate correlations that failed to obtain statistical significance (*p* > 0.05) are underlined.

**Table 2:** Moderation effects of behavioral coping (BC) on the relationship between perceived negative ageism (PNA) and several outcome variables.

Outcome	Predictor	*b*	95% CI	SE	*t*	beta
Quality of life		203.8 ***	[137.9, 269.7]	33.6	6.06	
	PNA	−14.4 ***	[−22.6, −6.25]	4.17	−3.46	−3.97
	BC	−3.48 *	[−6.57, −0.40]	1.57	−2.21	−1.34
	Age	−1.58 **	[−2.55, −0.61]	0.50	−3.19	−1.15
	PNA:BC	0.50 *	[0.11, 0.89]	0.20	2.52	3.16
	PNA:Age	0.18 **	[0.06, 0.30]	0.06	3.00	3.85
	BC:Age	0.06 *	[0.01, 0.10]	0.02	2.38	1.58
	PNA:BC:Age	−0.01 *	[−0.01, −0.00]	0.003	−2.32	−3.17
	*BC (lower)*, *55 yrs*	−3.18 ***	[−4.67, −1.70]	0.53	−6.01	−0.33
	*BC (higher)*, *55 yrs*	−0.23	[−1.63, 1.18]	0.50	−0.45	−0.02
	*BC (lower)*, *63 yrs*	−2.50 ***	[−3.08, −1.32]	0.31	−7.04	−0.23
	*BC (higher)*, *63 yrs*	−0.49	[−1.29, 0.30]	0.28	−1.74	−0.05
	*BC (lower)*, *68 yrs*	−1.59 ***	[−2.31, −0.87]	0.26	−6.20	−0.17
	*BC (higher)*, *68 yrs*	−0.66 *	[−1.32, −0.01]	0.23	−2.83	−0.07
	*BC (lower)*, *73 yrs*	−0.98 *	[−1.85, −0.11]	0.31	−3.15	−0.10
	*BC (higher)*, *73 yrs*	−0.83	[−1.67, 0.01]	0.30	−2.77	−0.09
	*BC (lower)*, *93 yrs*	1.47	[−1.12, 4.05]	0.92	1.59	0.15
	*BC (higher)*, *93 yrs*	−1.50	[−4.08, 1.07]	0.92	−1.64	−0.16
Mental well-being		52.2 ***	[47.1, 57.3]	2.58	20.2	
	PNA	−1.15 ***	[−1.69, −0.61]	0.28	−4.16	−0.51
	BC	0.08	[−0.12, 0.28]	0.10	0.77	0.05
	Age	0.07 ***	[0.04, 0.11]	0.02	3.85	0.09
	PNA:BC	0.03 **	[0.01, 0.06]	0.01	2.65	0.35
	*BC (lower)*	−0.84 ***	[−1.21, −0.47]	0.16	−5.15	−0.15
	*BC (higher)*	−0.05	[−0.39, 0.28]	0.15	−0.36	−0.01
Depression		13.11 ***	[9.17, 17.1]	2.01	6.52	
	PNA	0.97 ***	[0.55, 1.39]	0.21	4.53	0.57
	BC	0.10	[−0.05, 0.26]	0.08	1.31	0.08
	Age	−0.03 *	[−0.06, −0.00]	0.01	−2.21	−0.05
	PNA:BC	−0.03 **	[−0.05, −0.01]	0.01	−2.82	−0.38
	*BC (lower)*	0.72 ***	[0.43, 1.00]	0.13	5.65	0.16
	*BC (higher)*	0.07	[−0.19, 0.33]	0.12	0.58	0.01
Loneliness		5.76 ***	[3.24, 8.28]	1.29	4.48	
	PNA	0.45 **	[0.18, 0.72]	0.14	3.26	0.39
	BC	−0.19 ***	[−0.29, −0.09]	0.05	−3.85	−0.23
	Age	−0.01	[−0.03, 0.01]	0.01	−1.04	−0.02
	PNA:BC	−0.01	[−0.02, 0.00]	0.01	−1.52	−0.19

Note: *b* represents unstandardized regression weights or estimated marginal trends (for the simple slopes, following on significant interaction terms). Square brackets are used to enclose the lower and upper limits of a confidence interval (CI). The standard error (SE), *t*-value, and standardized regression weights (beta) are reported as well. * *p* < 0.05, ** *p* < 0.01, and *** *p* < 0.001. The *p*-values of the simple slopes are Bonferroni-corrected.

**Table 3:** Robustness check for confirmatory moderation analyses.

	Moderator	
	Behavioral Coping	Positive Appraisal Style
**Quality of life**		
No moderation	359	**868**
Two-way interaction	92	132
Three-way interaction with age	**549**	0
**Mental well-being**		
No moderation	291	**899**
Two-way interaction	**707**	97
Three-way interaction with age	2	4
**Depression**		
No moderation	187	468
Two-way interaction	**764**	**525**
Three-way interaction with age	49	7
**Loneliness (social, emotional)**		
No moderation	**808**	111 (347, 63)
Two-way interaction	94	**885** (244, **935**)
Three-way interaction with age	98	4 (409, 2)

Note: The number that should be the highest, based on the full-sample analysis, is shown in bold. Counts are out of 1000 bootstrap runs. We verified that the bootstrapping results were not biased by systematic differences (i.e., with some individuals systematically failing in the interaction categories and others systematically in the “no moderation” category).

**Table 4:** Moderation effects of a positive appraisal style (PAS) on the relationship between perceived negative ageism (PNA) and several outcome variables.

Outcome	Predictor	*b*	95% CI	SE	*t*	beta
Quality of life		108.75 ***	[105.1, 112.4]	1.88	58.0	
	PNA	−1.05 ***	[−1.19, −0.91]	0.07	−14.3	−0.29
	PAS	4.19 ***	[2.38, 6.00]	0.92	4.54	0.26
	Age	−0.09 ***	[−0.15, −0.04]	0.03	−3.43	−0.07
	PNA:PAS	0.16	[−0.07, 0.39]	0.12	1.39	0.08
Mental well-being		53.5 ***	[51.2, 55.9]	1.18	45.4	
	PNA	−0.42 ***	[−0.51, −0.33]	0.05	−9.22	−0.19
	PAS	3.03 ***	[1.90, 4.17]	0.58	5.24	0.31
	Age	0.08 ***	[0.04, 0.11]	0.02	4.31	0.09
	PNA:PAS	0.10	[−0.04, 0.25]	0.07	1.42	0.08
Depression		15.75 ***	[13.89, 17.61]	0.95	16.6	
	PNA	0.37 ***	[0.30, 0.45]	0.04	10.1	0.22
	PAS	−0.87 *	[−1.79, 0.04]	0.47	−1.88	−0.12
	Age	−0.04 **	[−0.07, −0.01]	0.01	−2.70	−0.06
	PNA:PAS	−0.13 *	[−0.25, −0.02]	0.06	−2.28	−0.14
	*PAS (lower)*	0.61 ***	[0.36, 0.87]	0.11	5.40	0.14
	*PAS (higher)*	0.14	[−0.10, 0.38]	0.11	1.33	0.03
Loneliness		0.71	[−0.55, 1.96]	0.64	1.11	
	PNA	0.26 ***	[0.21, 0.31]	0.02	10.4	0.23
	PAS	−0.28	[−0.89, 0.34]	0.31	−0.88	−0.05
	Age	0.00	[−0.02, 0.02]	0.01	0.18	0.00
	PNA:PAS	−0.12 **	[−0.20, −0.04]	0.04	−2.99	−0.19
	*PAS (lower)*	0.47 ***	[0.32, 0.62]	0.08	6.17	0.15
	*PAS (higher)*	0.05	[−0.09, 0.20]	0.07	0.76	0.02
Post hoc						
Social loneliness		−2.22	[−6.23 1.78]	2.04	1.09	
	PNA	0.55 *	[0.05, 1.05]	0.26	2.17	0.45
	PAS	4.41	[−1.65, 10.5]	3.09	1.42	−0.82
	Age	0.05	[−0.01, 0.10]	0.03	1.55	0.10
	PNA:PAS	−0.91 *	[−1.66, −0.16]	0.38	−2.38	−1.33
	PNA:Age	−0.00	[−0.01, 0.00]	0.00	−1.26	−0.29
	PAS:Age	−0.07	[−0.16, 0.02]	0.05	−1.59	−0.92
	PNA:PAS:Age	0.01 *	[0.00, 0.02]	0.01	2.13	1.21
	*PAS (lower)*, *55 yrs*	0.75 ***	[0.30, 1.21]	0.16	4.69	0.23
	*PAS (higher)*, *55 yrs*	−0.15	[−0.60, 0.31]	0.16	−0.90	−0.04
	*PAS (lower)*, *63 yrs*	0.55 ***	[0.27, 0.83]	0.10	5.50	0.17
	*PAS (higher)*, *63 yrs*	−0.02	[−0.29, 0.25]	0.10	−0.19	0.01
	*PAS (lower)*, *68 yrs*	0.42 ***	[0.19, 0.65]	0.08	5.10	0.13
	*PAS (higher)*, *68 yrs*	0.06	[−0.16, 0.28]	0.08	0.79	0.02
	*PAS (lower)*, *73 yrs*	0.29 *	[0.03, 0.56]	0.09	3.11	0.09
	*PAS (higher)*, *73 yrs*	0.14	[−0.11, 0.40]	0.09	1.55	0.04
	*PAS (lower)*, *93 yrs*	−0.22	[−0.95, 0.52]	0.26	−0.83	−0.07
	*PAS (higher)*, *93 yrs*	0.46	[−0.30, 1.22]	0.27	1.69	0.14
Emotional loneliness		0.91	[−0.34, 2.15]	0.63	1.43	
	PNA	0.24 ***	[0.19, 0.29]	0.02	9.68	0.21
	PAS	0.00	[−0.61, 0.61]	0.31	0.01	0.00
	Age	0.00	[−0.02, 0.01]	0.01	−0.49	−0.01
	PNA:PAS	0.13 **	[−0.20, −0.05]	0.04	−3.17	−0.20
	*PAS (lower)*	0.46	[0.29, 0.63]	0.08	6.09	0.15
	*PAS (higher)*	0.02	[−0.14, 0.18]	0.07	0.32	0.01

Note: *b* represents unstandardized regression weights or estimated marginal trends (for the simple slopes, following on significant interaction terms). Square brackets are used to enclose the lower and upper limits of a confidence interval (CI). The standard error (SE), *t*-value, and standardized regression weights (beta) are reported as well. * *p* < 0.05, ** *p* < 0.01, and *** *p* < 0.001. The *p*-values of the simple slopes are Bonferroni-corrected.

**Table 5:** Summary statistics and bivariate correlations with PNA per gender.

	Male			Female		
	M	SD	PNA	M	SD	PNA
**PNA**	7.37	2.58		7.41	2.67	
**MWB**	56.0	6.16	−0.16	55.6	5.75	−0.21
**QoL**	95.1	9.12	−0.28	94.6	9.82	−0.34
**DEP**	15.1	4.16	0.16	16.2 ***	4.64	0.24 *
**LONE**	2.68	2.90	0.23	2.72	3.13	0.24
**BC**	21.1	3.65		21.8 ***	3.68	
**PAS**	0.02	0.60		0.07	0.60	
**Age**	70.5 ***	7.08		67.2	6.67	

Note: PNA: perceived negative ageism; MWB: mental well-being; QoL: quality of life; DEP: depression; LONE: loneliness; BC: behavioral coping; PAS: positive appraisal style. Comparisons between the gender groups were made for each variable average and correlation coefficient. * *p* < 0.05 and *** *p* < 0.001.

**Table 6:** Exploratory (post hoc) analysis concerning the moderating role of gender.

	Moderator	
	Male (n = 665)	Female (n = 1334)
**Quality of life**		
No moderation	995	0
Two-way interaction with age	**5**	**1000**
**Mental well-being**		
No moderation	991	8
Two-way interaction with age	**9**	**992**
**Depression**		
No moderation	**1000**	**945**
Two-way interaction with age	0	55
**Loneliness**		
No moderation	**994**	**279**
Two-way interaction with age	6	721

Note: The number that should be the highest based on the full-sample analysis, is shown in bold. Counts are out of 1000 bootstrap runs, each containing n × 0.85 participants.

**Table 7:** Exploratory (post hoc) analysis concerning the effect of gender on the moderating roles of behavioral coping and a positive appraisal style.

	Behavioral Coping		Positive Appraisal Style	
Quality of life	Male	Female	Male	Female
No moderation	180	633	**1000**	**894**
Two-way interaction	0	355	0	106
Three-way interaction with age	**820**	**12**	0	0
**Mental well-being**				
No moderation	973	246	**999**	**756**
Two-way interaction	**13**	**748**	0	217
Three-way interaction with age	14	6	1	27
**Depression**				
No moderation	1000	9	957	672
Two-way interaction	**0**	**989**	**4**	**328**
Three-way interaction with age	0	2	39	0
**Loneliness (social, emotional)**				
No moderation	**975**	**985**	724 (933, 501)	363 (667, 303)
Two-way interaction	9	13	**276** (19, **478**)	**636** (151, **697**)
Three-way interaction with age	16	2	0 (**48**, 21)	1 (**182**, 0)

Note: The number that should be the highest, based on the full-sample analysis, is shown in bold. Counts are out of 1000 bootstrap runs, each containing n × 0.85 participants.

## Data Availability

Pre-registered on 16 August 2023: https://osf.io/4sbac. The datasets presented in this article are not readily available because the datasets used and/or analyzed for the current study will only be made publicly available after the completion of the overarching project and will, until that time, only be available from the corresponding author on a collaboration basis upon reasonable request. Requests to access the datasets should be directed to L.B. (l.p.brinkhof@uva.nl).

## Supplementary Materials

The following supporting information can be downloaded at https://www.mdpi.com/article/10.3390/geriatrics9010001/s1, Supplement S1, Exploration of Other Resilience Factors; Supplement S2, Moderation Effects of Age.
