# Supplementary material for: Mitigating the Harmful Impact of Ageism among Older Individuals: The Buffering Role of Resilience Factors
Source: Geriatrics (Basel). 2023 Dec 20;9(1):1. doi: 10.3390/geriatrics9010001 (PMC10888260; doi:10.3390/geriatrics9010001)
Supplement: Supplementary file 1 [file geriatrics-09-00001-s001.zip › Supplement S1.pdf]

Supplement A: Mitigating the Harmful Impact of Ageism Among Older Individuals: the Buffering Role of Resilience Factors.

This supplement reports the exploration of several other resilience-related factors as potential buffers of the detrimental impact of perceived negative ageism on quality of life, mental well-being, depression and loneliness.

Background

In addition to two coping-related factors (i.e., behavioral coping and positive appraisal style; see main text), which are thought to be instrumental to an individual's ability to adapt and rebound from adversity, we also explored some other resilience-related factors as potential buffers of the adverse relationship between perceived negative ageism and our outcome variables of interest. These include: self-efficacy, self-esteem, social participation, and the big-five personality traits.

The first factor that may contribute to better QoL and mental well-being/health in the face of negative ageist encounters is *self-efficacy* (i.e., a person's belief in his/her ability to execute behaviors necessary to obtain a certain outcome; [54–56]). Frequent exposure to ageism is known to lower individuals' self-efficacy, thereby negatively affecting (mental) health [57,58]. However, high levels of self-efficacy prior to an ageist encounter may also protect against the negative consequences of ageism. High levels of self-efficacy could help individuals to prevent self-doubts, cope with the elicited threat that getting older creates barriers to one's goals and wellbeing, and incite active deliberation about ways in which age-related changes could be dealt with.

Self-esteem constitutes another psycho-social factor that could mitigate the negative effects of perceived negative ageism. It reflects how a person feels about themselves, and can be considered as a general evaluation of self-worth. Bergman [18] found that the ageism-psychological distress link was only significant among individuals with low self-esteem and low positive body image, suggesting that holding a positive self-esteem and/or a positive perception of one's body may jointly buffer the connection between ageism and psychological distress in older adults. Whether high levels of self-esteem may provide a buffer independently and has similar implications for individuals' quality of life and mental well-being, remains to be established.

Having various (worthwhile) social contacts may also act as effective strategy to ward against the negative effects of perceived ageism. Social participation is a well-known protective factor for successful aging (e.g., [63 - 65]), and has been identified as important predictor of individuals' attitudes towards aging, thereby reducing the risk of mental health problems in later life [66]. Previous research has shown that social participation moderated the effects of negative age stereotype priming

on episodic memory [67]. However, there is a dearth of research on the role of social participation in bolstering resilience against the adverse effects of perceived ageism on quality of life and mental well-being/health. Findings concerning social support underscore the potential of this factor [68–70]: social support has been documented to act as a buffer against the impact of perceived discrimination, whether it is based on race or age, on psychological distress and other relevant outcome variables, such as life satisfaction. Indeed, being actively involved in social activities can lead to the development of stronger social bonds and relationships, and social participation therefore often serves as a means to access social support.

Lastly, it is also worth exploring individual differences in the big-five personality traits as potential factors that may determine whether individuals are affected by ageism or not. Openness, conscientiousness, agreeableness, emotional stability and extraversion are all positively correlated with successful aging and resilience [71,72]. As of yet, no previous research has investigated their buffering potential in relation to the link between ageism and quality of life, mental well-being/health (see [73] for a study on workplace discrimination). We contemplate the following expectations [74]. Individuals that are high in openness to experience are more receptive to new perspective and ideas, have a strong desire to learn and acquire knowledge, and tend to be more willing to listen to multiple viewpoints. Consequently, they may be more inclined to engage in conversations to challenge other’s age-related biases, which may prove helpful in mitigating its negative effects. However, this openness can also render them more receptive to absorbing negative sentiments, potentially leading to greater negative consequences in the long-run. Conscientiousness individuals tend to be better at planning for the future, including their own aging process. Their organized and responsible nature may help them effectively cope with ageism-related stressors and find constructive ways to address ageism. Extraverted individuals often have larger social networks and more robust social support systems. These connections may provide instrumental or emotional buffering against the negative effects of ageism. Agreeable individuals are often adept at conflict avoidance and prioritize nurturing harmonious relationships, traits that frequently translate into larger and more supportive social networks. This can facilitate positive and constructive interactions when confronted with ageism, potentially benefiting one's psychological and physical well-being. However, in the context of ageism, their preference for harmony may lead to tolerating or disregarding ageist behaviour, even if they are hurtful or discriminatory, hesitating to assert themselves and suppressing their genuine feelings. Such responses may heighten emotional distress, foster stereotype internalization, and (thereby) negatively impact long-term quality of life and mental well-being/health. Finally, individuals low in neuroticism and high in emotional stability, are less prone to experiencing intense negative emotions in response to stressful situations. Hence, they may be more resilient in the face of ageism as well.

Materials

General Self-efficacy

The Dutch General Self-Efficacy Scale is used to assess how someone generally copes with stressors or challenging situations in life [59,60] (α = 0.88). The scale concerns ten statements that ask how people generally think and act, focusing explicitly on a person's self-confidence that his or her actions are responsible for successful outcomes or that they have control over challenging demands of the environment. Each statement is scored on a 4-point Likert Scale (1 = not true at all, 2 = hardly true, 3 = moderately true, 4 = exactly true) and summed to a total score of 10 to 40. A higher score reflects higher general self-efficacy.

Self-esteem

The Rosenberg Self-esteem Scale was used to assess global self-esteem [61,62] (α = 0.84). The scale includes 10 items (e.g.,) that are scored on a 4-point Likert scale (1 = totally disagree, 2 = disagree, 3 = agree, 4 = totally agree). After reverse scoring some of the items, all items were summed to a total of 0 to 40, with higher scored indicating a higher global self-esteem.

Social participation

A social participation list (α = 0.69) was generated to assess engagement in social activities over the past year. The list includes eight different activities (e.g., meeting with friends, participating in a competitive sport, going to a concert with others) and participants are asked to report the number of times they had done that activity with someone else in the past year based on the following categories: 0 = not this year, 1 = 1-5 times in the past year, 2 = at least once every two months, 3 = once every 2 or 3 weeks, 4 = once a week, 5 = more than once a week. A total participation score was calculated by aggregating all items (0-40), with higher scores indicating more frequent engagement in social activities over the previous year.

Personality

The Ten Item Personality (TIPI) measure was used to assess the Big Five personality dimensions: extraversion, agreeableness, conscientiousness, neuroticism, openness 75,76] (α – 0.60). The scale consists of 10 pairs of characteristics (e.g., extraverted, enthusiastic; anxious, easily upset)/ Each pair was rated on a scale from 1 – 7 (with 1 = does not describe me at all and 7 = describes me very well) and reverse scored if appropriate. An average score per domain was calculated. A higher score for its respective domain reflect a higher level of extraversion, agreeableness, conscientiousness and openness and a lower level of neuroticism (thus a higher level of emotional stability).

Although the Dutch version had been validated and was (back-)translated by a native expert and two independent researchers, we believe that the pair 'critical, quarrelsome' has been incorrectly translated. Being critical might be primarily seen as a negative thing in the English language (i.e., expressing adverse or disapproving comments or judgements), but in the Dutch language it is easily confused with critical thinking, which is seen as a very valuable trait in many circumstances. Hence, we adapted the Dutch translation to 'veroordelend' (i.e., judgmental).

Exploratory analysis

We used a bootstrapping analysis to explore the potential of the selected resilience factors. We randomly selected 500 participants from the full sample (N = 2000) and temporarily excluded them, creating a new subsample. Subsequently, we subjected the remaining dataset (N = 1500) to the moderation analysis, as described in the main article. This bootstrapping process was repeated 1000 times, generating a multitude of subsamples and conducting moderation analyses for each. By doing so, we could assess how frequently a two-way interaction, three-way interaction, or no moderation effect was observed (using an alpha level of 0.05), and thereby obtain an indication of the potential buffering effect of these resilience factors.

Results

The results are presented in Table SA1 and Table SA2. None of the explored factors appeared to be a convincing resilience factor for all outcome variables of interest, with many factors having high incidences of 'no moderation'. This suggests that, overall, the effects of negative ageism remain largely exempt from moderation by more distal resilience factors.

Nonetheless, we found some evidence to believe that it may be worthwhile to further evaluate the potential of self-efficacy, self-esteem, social-participation and various personality traits as buffering factors in relation to specific outcome variables. Self-efficacy may protect individuals from being emotionally lonely when perceiving high levels of negative ageism; self-esteem seemed to provide a buffer for depression, overall loneliness and social loneliness in particular. Social participation may mitigate the negative effects of PNA on depression only. Lower scores on the personality trait neuroticism (i.e., being more emotional stable) seemed to mitigate the PNA-depression link, independent of age. Higher levels of conscientiousness and extraversion may protect relatively younger individuals from feelings of loneliness (overall, emotional and, in case of conscientiousness, social). Higher scores on extraversion may also be beneficial to maintain mental well-being when frequently exposed to ageism. The same is true for openness, although this seems

most likely to apply to relatively younger individuals only. Finally, extraversion may also provide a buffering effect for relatively younger individuals' quality of life.

While relatively low counts for 'no moderation' were found for openness and agreeableness in relation to QoL, suggesting these personality traits may also provide a buffer for individuals' QoL, inspection of the slopes (either with or without age being taken into account) indicates that higher scores on these traits may actually be less beneficial.

References

[18] Bergman YS. Ageism and Psychological Distress in Older Adults: The Moderating Role of Self-Esteem and Body Image. J Appl Gerontol 2022;41:836–41. https://doi.org/10.1177/07334648211009658.

[54] Bandura A. Self-efficacy: Toward a unifying theory of behavioral change. Psychol Rev 1977;84:191–215. https://doi.org/10.1037/0033-295X.84.2.191.

[55] Bandura A. Social Foundations of Thought and Action: A Social-Cognitive View. NJ: Englewood Cliffs; 1986.

[56] Bandura A. Self-efficacy: The exercise of control. New York: W.H. Freeman; 1997.

[57] Chang ES, Kannoth S, Levy S, Wang SY, Lee JE, Levy BR. Global reach of ageism on older persons' health: A systematic review. PLoS One 2020;15:1–24. https://doi.org/10.1371/journal.pone.0220857.

[58] Tovel H, Carmel S, Raveis VH. Relationships Among Self-perception of Aging, Physical Functioning, and Self-efficacy in Late Life. Journals Gerontol Ser B 2019;74:212–21. https://doi.org/10.1093/GERONB/GBX056.

[59] Schwarzer R, Jerusalem M. General Self--efficacy Scale (GES). 1995.

[60] Teeuw B, Schwarzer R, Jerusalem M. Dutch adaptation of the general self-efficacy scale. Berlin, Ger 1994.

[61] Franck E, De Raedt R, Barbez C, Rosseel Y. Psychometric properties of the Dutch Rosenberg self-esteem scale. Psychol Belg 2008;48:25–35. https://doi.org/10.5334/pb-48-1-25.

[62] Rosenberg M. Conceiving the self. New York: Basic Books; 1979.

[63] Chen J, Zeng Y, Fang Y. Effects of social participation patterns and living arrangement on mental health of Chinese older adults: A latent class analysis. Front Public Heal 2022;10:915541. https://doi.org/10.3389/FPUBH.2022.915541/BIBTEX.

[64] Ibrahim AF, Tan MP, Teoh GK, Muda SM, Chong MC. Health Benefits of Social Participation Interventions among Community-Dwelling Older Persons: A Review Article. Exp Aging Res 2022;48:234–60. https://doi.org/10.1080/0361073X.2021.1939563.

[65] Wanchai A, Phrompayak D. Social Participation Types and Benefits on Health Outcomes for Elder People: a Systematic Review. Ageing Int 2019;44:223–33. https://doi.org/10.1007/S12126-018-9338-6/TABLES/1.

[66] Mao S, Zeng Y, Lu N. Research on Attitudes towards Ageing, Social Participation and Depressive Symptoms Among Older Adults in China. Br J Soc Work 2023;53:698–717. https://doi.org/10.1093/BJSW/BCAC118.

[67] Chan SCY, Au AML, Lai SMK. The detrimental impacts of negative age stereotypes on the episodic memory of older adults: does social participation moderate the effects? BMC Geriatr 2020;20:1–12. https://doi.org/10.1186/S12877-020-01833-Z/FIGURES/4.

[68] Steers MLN, Chen TA, Neisler J, Obasi EM, McNeill LH, Reitzel LR. The buffering effect of social support on the relationship between discrimination and psychological distress among church-going African-American adults. Behav Res Ther 2019;115:121–8. https://doi.org/10.1016/j.brat.2018.10.008.

[69] Ajrouch KJ, Reisine S, Lim S, Sohn W, Ismail A. Perceived everyday discrimination and psychological distress: Does social support matter? Ethn Heal 2010;15:417–34. https://doi.org/10.1080/13557858.2010.484050.

[70] Redman T, Snape E. The consequences of perceived age discrimination amongst older police officers: Is social support a buffer? Br J Manag 2006;17:167–75. https://doi.org/10.1111/j.1467-8551.2006.00492.x.

[71] Baek Y, Martin P, Siegler IC, Davey A, Poon LW. Personality Traits and Successful Aging: Findings from the Georgia Centenarian Study. Int J Aging Hum Dev 2016;83:207–27.

https://doi.org/10.1177/0091415016652404.
[72] Oshio A, Taku K, Hirano M, Saeed G. Resilience and Big Five personality traits: A meta-analysis. Pers Individ Dif 2018;127:54–60. https://doi.org/10.1016/j.paid.2018.01.048.
[73] Xu YE, Chopik WJ. Identifying Moderators in the Link Between Workplace Discrimination and Health/Well-Being. Front Psychol 2020;11:488768. https://doi.org/10.3389/fpsyg.2020.00458.
[74] Costa PT, McCrae RR. Revised NEO Personality Inventory (NEO PI-R) and NEO Five-Factor Inventory (NEO-FFI) professional manual. Psychological Assessment Resources; 1992.
[75] Gosling SD, Rentfrow PJ, Swann WB. A very brief measure of the Big-Five personality domains. J Res Pers 2003;37:504–28. https://doi.org/10.1016/S0092-6566(03)00046-1.
[76] Hofmans J, Kuppens P, Allik J. Is short in length short in content? An examination of the domain representation of the Ten Item Personality Inventory scales in Dutch language. Pers Individ Dif 2008;45:750–5. https://doi.org/10.1016/j.paid.2008.08.004.

Table S1

Summary statistics and associations per gender

	Male		Female		PNA	MWB	QoL	DEP	LONE	GSE	SE	SP	CON	OPE	EMO	AGR
	**M**	**SD**	**M**	**SD**												
**Perceived negative ageism**	7.37	2.58	7.41	2.67												
**Mental well-being**	56.0	6.16	55.6	5.75	-0.19											
**Quality of life**	95.1	9.12	94.6	9.82	-0.32	0.62										
**Depression**	15.1	4.16	16.2***	4.64	0.22	-0.63	-0.61									
**Loneliness**	2.68	2.90	2.72	3.13	0.24	-0.44	-0.59	0.45								
**Self-efficacy (GSE)**	32.9	4.18	32.56	4.13	-0.14	0.53	0.44	-0.38	-0.26							
**Self-esteem (SE)**	33.4*	4.01	32.9	4.20	-0.27	0.57	0.55	-0.47	-0.38	0.50						
**Social participation (SP)**	11.3	6.43	14.2**	6.34	-0.06	0.18	0.22	-0.12	-0.27	0.14	0.12					
**Conscientiousness (CON)**	10.8	2.12	11.1**	2.12	-0.09	0.20	0.17	-0.14	-0.08	0.22	0.25	-0.01				
**Openness (OPE)**	10.8	2.32	11.1***	2.36	-0.02	0.23	0.16	-0.07	-0.10	0.29	0.26	0.12	0.10			
**Emotional stability (EMO)**	11.3***	2.02	10.7	2.32	-0.15	0.43	0.37	-0.39	-0.24	0.47	0.48	0.10	0.17	0.15		
**Agreeableness (AGR)**	11.7	1.59	11.9**	1.61	-0.18	0.29	0.26	-0.15	-0.23	0.21	0.34	0.04	0.21	0.18	0.27	
**Extraversion (EXT)**	9.26	2.72	10.1***	2.73	-0.10	0.26	0.22	-0.17	-0.23	0.21	0.28	0.20	0.07	0.30	0.07	0.12

Note. PNA: perceived negative ageism, MWB: mental well-being, QoL: quality of life, DEP: depression, LONE: loneliness. The bivariate correlations that failed to obtain statistical significance ($p > .05$) are underlined. *$p < .05$, **$p < .01$, ***$p < .001$

Table S2

Exploring other potential moderators

	Potential moderator							
	**Self-efficacy**	**Self-esteem**	**Social Participation**	**Conscientiousness**	**Openness**	**Emotional stability**	**Agreeableness**	**Extraversion**
**Quality of life**								
No moderation	992	818	841	910	407	939	316	169
Two-way interaction	8	19	158	48	234	5	264	218
Three-way interaction with age	0	163	1	42	359	56	420	**613**
**Mental well-being**								
No moderation	653	863	838	954	435	971	991	423
Two-way interaction	0	11	133	1	3	25	9	**344**
Three-way interaction with age	347	126	29	45	**562**	4	0	**233**
**Depression**								
No moderation	999	311	442	993	984	370	987	646
Two-way interaction	1	**689**	**557**	0	16	**629**	13	348
Three-way interaction with age	0	0	1	7	0	1	0	6
**Loneliness (Social, Emotional)**								
No moderation	530 (812, 430)	411 (314, 674)	996 (950, 994)	165 (281, 349)	953 (937, 852)	973 (847, 986)	790 (768, 505)	488 (932, 271)
Two-way interaction	419 (83, **556**)	**576** (**474**, 325)	4 (13, 6)	0 (1, 17)	1 (14, 108)	4 (4, 12)	55 (1, 445)	3 (18, 0)
Three-way, with age	51 (105, 14)	13 (**212**, 1)	0 (37, 0)	**835** (**718, 634**)	46 (49, 40)	23 (149, 2)	155 (231, 50)	**509** (50, **729**)

Note. Counts that suggest their may be a buffering effect of the corresponding factor are shown in bold. For the factors where the simple slopes suggest negative implications, the counts are underlined.
