# Supplementary material for: Mitigating the Harmful Impact of Ageism among Older Individuals: The Buffering Role of Resilience Factors
Source: Geriatrics (Basel). 2023 Dec 20;9(1):1. doi: 10.3390/geriatrics9010001 (PMC10888260; doi:10.3390/geriatrics9010001)
Supplement: Supplementary file 1 [file geriatrics-09-00001-s001.zip › Supplement S2.pdf]

Supplement B: Mitigating the Harmful Impact of Ageism Among Older Individuals: the Buffering Role of Resilience Factors.

Table S3

Moderation Effects of the Relationship between Perceived Negative Ageism and Several Outcome Variables

Outcome	Predictor	*b*	95% CI	SE	*t*	beta
Quality of life		135.5***	[123.9, 147.1]	5.92	22.9	
	PNA	-4.23***	[-5.68, -2.78]	0.74	-5.72	-1.16
	Age	-0.48***	[-0.65, -0.31]	0.09	-5.51	-0.35
	PNA:Age	0.05***	[0.02, 0.07]	0.01	4.26	0.95
Mental well-being		67.03***	[59.6, 74.5]	3.79	17.7	
	PNA	-1.95***	[-2.88, -1.03]	0.47	-4.13	-0.88
	Age	-0.12*	[-0.22, -0.01]	0.06	-2.09	-0.14
	PNA:Age	0.02**	[0.01, 0.03]	0.01	3.18	0.74
Depression		11.87***	[6.18, 17.6]	2.90	4.09	
	PNA	0.77*	[0.06, 1.48]	0.36	2.12	0.45
	Age	0.02	[-0.07, 0.10]	0.04	0.38	0.02
	PNA:Age	-0.01	[-0.02, 0.00]	0.01	-1.06	-0.25
Loneliness		-2.71	[-6.54, 1.12]	1.95	-1.39	
	PNA	0.64**	[0.16, 1.12]	0.24	2.64	0.56
	Age	0.05	[-0.01, 0.11]	0.03	1.76	0.11
	PNA:Age	-0.01	[-0.01, 0.00]	0.00	-1.55	-0.36

Note. b represents unstandardized regression weights or estimated marginal trends (for the simple slopes, following on significant interaction terms). Square brackets are used to enclose the lower and upper limits of a confidence interval (CI), and both the standard error (SE) and t-value are reported. **p* < .05, ***p* < .01, ****p* < .001. In case the moderation term is insignificant, the main effect should not be interpreted. Please see Table 1 (main text) for the bivariate correlation.
